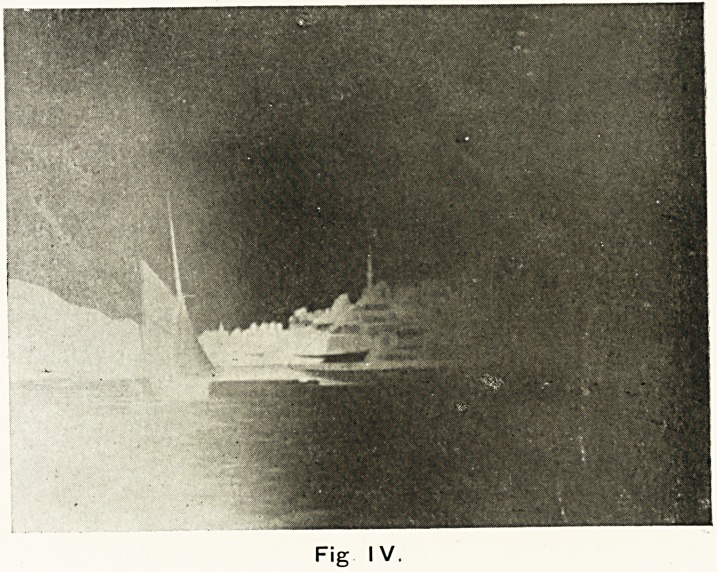# Some Remarks upon the Finsen Light Treatment of Lupus

**Published:** 1903-06

**Authors:** W. Kenneth Wills


### SOME REMARKS UPON THE FINSEN LIGHT TREATMENT OF LUPUS.

BY

W. KENNETH WILLS, MA., M.B., B.C. (Cantab.), M.R.C.S.

To Professor Finsen, of Copenhagen, is due the credit of introducing to the medical world the possibilities of treating successfully diseases of the integument by light. It very possibly had not occurred to him how great a field he was opening up when first he directed his attention to concentrating the radiant energy of the sun upon the skin for the purpose of destroying *in situ* the germs of disease; but undoubtedly he was the pioneer of all that is now included in the term Photo-therapy.[1]

In the first place he had noticed some deleterious results of the chemical rays of light—those situated at the violet end of the spectrum, and including the green, blue, violet, and especially the ultra-violet rays—upon the skin of man and of lower animals. His observations upon small-pox led him to consider the advisability of excluding these chemical rays from the room of a small-pox patient; and this resulted in preventing, for the most part, suppuration and pock-marking in the majority of these cases.

His next observation was the fact that a large number of the lower forms of animal life show a marked distaste for chemical rays. He found that this was due to an uncomfortable excitation produced when these rays fell upon their bodies, and he determined that nature had provided against deleterious exposures to such rays by causing a pigmentary deposit in the skin of these creatures chronically exposed to the rays of the sun. That this was not due to heat he proves by instancing the pigmentation of fish who, living under water, cannot be affected to any large degree by the sun's heat, and to the fact that at temperatures below zero mountain-climbers can experience high degrees of "sunburn," which end in pigmentation of the skin. He concludes from these data that the chemical rays are deleterious.

[1] *Photo-therapy*: Finsen. Translated by Sequeira, 1901.

However, further investigations showed him that light had an exhilarating effect, which he attributes to an excitement of the nervous system. This he found could be turned to good account, and he tried the exhilarating effect of general light baths in cases of debility with some success. The immediate result of such baths, even in the cold, was to produce an erythema which was not developed immediately, such as a heat reaction would be, but had some latent period, and the effect lasted for some time after the application had ceased.

He then attempted the treatment of skin disease by means of concentrated light, with the idea that, the chemical rays being deleterious to the lower forms of living matter, the germs producing such disease might be killed. Various forms of dermatoses known to be produced by micro-organisms were treated, and especially lupus vulgaris, which being produced by the bacillus tuberculosis, a germ known to be killed by light, lent itself as a test disease for such a treatment.

The bactericidal properties of light are very marked, but they are slow, hence it became necessary to cut off the heat rays from the sunlight, which was used at first to prevent heat reactions.

At first a hollow convex lens filled with a solution of methyline blue was used, which effectually cut off the yellow, red, and ultra-red rays. This gave place to a telescopic adjustment with a deeper water chamber. Latterly, the electric light has been used as being always obtainable, more easily handled, and richer in chemical rays.

The time of exposure was at first about two hours; but better apparatus, including the substitution of lenses made of quartz for glass, which cuts off a large amount of the ultra-violet rays, has led to the reduction of the exposure by Finsen's apparatus to one hour.

The original apparatus consists of a central powerful arc lamp, of 50 to 60 ampères, from which radiate telescopic condensing tubes, in which water is kept circulating.

The patient is in such a position that the light can be focussed upon the part treated, and this is compressed by means of a quartz cell, in which again water is kept circulating.

The tissues are all to a greater or less degree permeable by light. This has been proved by many experiments. Light has been passed through the body of a well-made man, and the fore-arm bones photographed upon a plate by the aid of light alone (Gottheil and Franklin).[1] But all the rays of light are not equally able to pass through. The red and yellow rays are more transmissible than the blue or violet. Indeed, blood absorbs the chemical rays so readily that they penetrate a very short distance in the tissues. Hence it is necessary to express the blood from the part treated before the chemical rays are able in any numbers to reach the diseased part.

Finsen found that the strong light of his focussing apparatus was insufficient to influence photographically sensitive paper placed behind the ear in five minutes, until the ear was compressed between two glasses and so rendered exsanguine, when blackening ensued in twenty seconds.

As mentioned above, glass is not suitable as a compressor, for an electric arc of 1,200 candle power has failed to affect the skin when it was filtered through glass (Widmark). Water and ice, while effectually cutting off the heat rays, allow the violet and ultra-violet rays to pass. Considering that these rays so predominate in water, it has occurred to me that the pigmented lateral line of fishes, endowed as it is with a trunk nerve, may be an organ of sense by which fish may be able to determine how much they are exposed to direct sunlight, and so escape too much exposure to the keen sight of enemies. Ice is a useful compressor, as it acts by means of the cold in driving out the blood from the vessels.

After an exposure to this chemical light, a "reaction" of greater or less severity ensues. This intensity of reaction depends upon several factors: the dosage of the light, the translucency of the part treated, the amount of pigment, and the individuality of the patient. In its slight degree the "reaction" consists of an erythema, with its attendant itching and burning, which fades away in a day or two, leaving a powdering of the epithelium. In its greater degree there is a vesicle formation more or less superficial, which gradually dries up and sheds its epithelium in large flakes. Some reaction is essential to the

[1] *Med. Rec.*, 1902, lxi. 609.

beneficial effect of the treatment, and the deeper seated the disease, the deeper the reaction must reach. What actually brings about the good effect, it is impossible as yet to say. Inflammation, oxygenation, nervous stimulation, and bactericidal action no doubt all assist in it. It cannot be the mere inflammation of an antiseptic, else the application of inflammatory antiseptics would bring about a similar result.

The ultimate result of even a deep-seated erythema is merely pigmentation: scarring is not produced; and the treatment can be repeated again and again without producing any untoward results. The usual method adopted is to treat continuously one or more spots, repeating the applications until the nodules of lupus, distinguished by the light-brown, freckle-coloured "apple-jelly" deposit, have entirely disappeared.

In this way the disease is eradicated, care being taken to treat a growing margin at first, and when the growth is checked the attention is paid to the other parts in order. Not only is there a good effect upon active disease, but also scar-tissue is rendered more supple and elastic. It tends less and less to bind down the skin and lead to such hideous contortions and deformities.

A phenomenon I have remarked is noteworthy. When a patient has come under treatment with the lupus spreading rapidly, when part of the growing margin has been treated, the extension in other directions seems at times to be simultaneously arrested: and parts not treated in other positions in the body occasionally share the improvement in the part treated, though not of course in the same degree.

After a patch of lupus has been treated continuously for some time and the reaction has been allowed to subside, the apple-jelly deposits are found to have become paler in colour, and these gradually disappear entirely under treatment. It is a matter of some experience to tell when a patch is quite healed, and this difficulty accounts for the number of cases which return for further treatment after they have been allowed to discontinue the treatment owing to the disappearance of the deposits. The so-called recurrences are, it is supposed, in reality foci which were not completely cured, and these yield to treatment eventually. The scar left is in some ways different from that produced by operations. It is not so white, being more the colour of the surrounding skin, and it is more supple, not leading to contraction.

There are some patients who do not react readily to the light, and who therefore require very long exposures. For these cases it is useful to use some application which will irritate the skin somewhat, after which much shorter exposures will be found sufficient. Various substances have been found to be useful in this way, notably pyrogallol and carbolic acid. It is useful, and in some cases necessary, to use a solution of caustic potash to remove the scales, and ether to remove any grease which may be present. These will act in the same way, but one objection to the use of any of these substances is that the hyperæmia produced has to be combatted by greater pressure to render the parts exsanguine.

Other agencies comprise: Acid nitrate of mercury; several consecutive applications of light to one spot (Dore)[1]; one per cent. solution of permanganate of potash; a solution of iodine and glacial acetic acid (viz.: iodine 1, pot. iod. 2, glacial acetic acid 2, water 100.) Macleod.[2]

Personally, I find that an occasional exposure to the X-rays will increase the reactions from the light considerably, and this effect is not lost so easily. The X-rays are useful as an adjunct in other ways: they are beneficial in healing ulcerations as well as reaching parts which cannot be treated by the light. For instance, in a boy, aged 13, the light was used successfully at first for lupus involving the alæ, and tip of the nose, and the upper lip: the septum of the nose was ulcerated away, and a deep ulcer extended from the lip up into the nostrils, the discharge from which completely occluded both nostrils with thick crusts. It was impossible to reach this ulcer effectually with the smallest pressure-glass of the lamp, but the X-rays, after twenty exposures, have healed the whole completely, leaving a clean, and what appears to be a sound, scar behind.

The X-rays have a peculiar selective affinity for lupus. In the case of a girl, aged 16, with deep-seated extensive lupus on the right side of the face and neck, a slight overdose of the rays

[1] Dore, *Brit. M. J.*, 1902, ii. 1317. [2] Macleod, *Ibid.*

produced a small "burn" on the diseased part exposed, while the normal skin showed merely an erythema.

Some cases of lupus show considerable thickening and infiltration of the parts. For this condition pyrogallol is recommended by Dore and Macleod,[1] and the internal use of thyroid extract. In one case where I used the latter, in the hope of reducing a great hypertrophy of the upper lip in a man aged 21, the lip certainly became much smaller and he himself much thinner; but I could not determine how much the mere pressure of the lens had to do with the lessening of the size of the lip.

The œdema caused by the light reaction is in some patients considerable, especially in the region round the orbit, where the cellular tissue is loose. The eye should in all cases be efficiently protected, as a very painful conjunctivitis may ensue from undue exposure. A striking instance of this occurred in my own consulting room, where an electrician's assistant was holding an iron-electrode lamp (Leslie Miller) while his mate was fitting resistance so as to work it from the mains. After it had been working for a few hours with intermittent doses of ultra-violet light, he had to spend the rest of the day bathing his eyes with cold water. He was by no means well by the next morning.

The Tesla high-frequency currents have been used to increase reaction, as well as in the complete treatment of lupus. My experience as yet is too limited to say more than that in one case considerable exfoliation followed a few minutes' application with a pointed glass electrode to the margins of the nostrils, and a deficiency in the septum where the disease was still active. In another case, in which the whole of the face was hide-bound by scarring, after a few treatments with a glass electrode the patient stated that her face felt less drawn, though I could not personally detect any difference by touch.

Some cases of lupus seem to resist the rays. What these are cannot at present be foretold. Some of the oldest cases at the Bristol General Hospital are improving, while others more recent do not seem so hopeful. As a rule, however, the more recent the disease the more readily it reacts.

[1] Dore, *loc. cit.*

Scarring undoubtedly protracts the treatment very considerably. The best cases for treatment are those where the disease is recent, and where no surgical procedure has been carried out, either by knife, cautery, or caustics.

A word about the various forms of lamps in use at the present day.

It is not in the power of everyone to obtain the enormous plant required for the carrying out of the treatment exactly as Finsen himself does. But equally good results can be obtained by smaller installations. The essentials in a lamp are: Its light must be—

(1) Intense,
(2) Rich in chemical rays,
(3) Cool, and
(4) It must penetrate the tissues.

It is not as yet ascertained with any degree of exactitude what are the most important rays in photo-therapeutics. It is possible, if not indeed probable, that the shorter the wave length the more useful the rays are; the longer they are, to a certain point, the deeper they penetrate. The rays in between the two extremes would therefore seem to be the most advantageous as possessing both these characteristics to a certain extent, and these include the extreme violet and blue. But I think, as has been suggested I believe by Dr. Ernest Dore, there is evidence to show that the waves of longer length in their penetration carry with them shorter-lengthened rays, which would not themselves penetrate deeply.

Finsen himself used the white light of the electric arc, but Bangs, a colleague of his at Copenhagen, suggested the use of an arc lamp with iron electrodes instead of carbons. The resulting light is a bright violet in colour, and although not nearly so intense to the eye as a carbon arc, is richer by far in violet and ultra-violet rays. The eye being only able to detect wave lengths of about 392 $\mu. \mu.$ (millionths of a millimetre) the rays giving shorter wave lengths than this cannot be appreciated, though a photographic plate is affected. The wave length can be raised in length by various fluorescent substances, such as the alkaline earths, the platino-cyanide of barium screen, or willemite, a silicate of zinc, esculine, fluorescine, sulphate of quinine, and lead glass. These substances fluoresce brilliantly under the "iron light."

The best known lamps made on this principle are the "Dermo," with water-cooled iron electrodes, and the St. Bartholomew's Hospital lamp (Leslie Miller). I have had two of the latter in use at the Bristol General Hospital, and can testify to the fact that good results may be obtained by their means; but the good is not by any means proportional to the relative richness in chemical rays, and the reason of this must be sought in the want of penetration.

Several smaller carbon lamps are now being used: Marshall and Wood's, with a water chamber through which the light passes and is cooled in the metal case—a modification of the lamp of Lortet and Genoud ("French lamp"); the Cox lupus lamp (Heathcote patent); and the new Danish lamp (Bang's), both with water-cooled carbons. These all are of advantage in being practically smaller, and therefore cheaper editions of the Finsen lamp.

I have had made for me, with the skilled assistance of Mr. A. Pitt, a lamp combining the advantages of both the carbon and iron electrodes. The iron is so arranged as to yield its vapour in the burning of the carbons, and in this way is produced a light which is very rich in the ultra-violet rays, and is also rich in the rays of more penetrating power. It is fitted with a running water chamber, which cools the light effectually, and is very portable. When used with a set-up transformer and a condenser, the alternating electric main supply can be utilised, and a very small amount of current is used, about ½ amp. Although I have not had time to do more than a few simple experiments with this lamp as yet, enough has been done to show that it contrasts favourably with the Marshall and Wood's and the St. Bartholomew's lamps. The reaction upon my own arm has been greater under similar conditions. Reactions upon patients have been much more pronounced, and, as far as can be seen, deeper. The fluorescent effects have been increased from a distance of three feet with the St. Bartholomew's lamp, to nine feet. The penetrating effects, as shown by photographic plates bound to the back of a thin boy, have

Fig. I.

Fig. II.

Fig. III.

Fig IV.

been somewhat greater than with the Marshall and Wood's carbon lamp.

Freund has determined spectroscopically that chemically active rays pass freely through the epidermic tissues of considerable thickness, and if it is a true assumption that white light will aid the penetration of the ultra-violet rays, then we should in this combination add considerably to our armamentarium. Since my experiments were commenced, much the same idea has been utilised in a new lamp brought out by the Sanitas Company, called the "Triplet." In this one iron and one carbon electrode can be used together for the production of "mixed" light. This arrangement does not promise so intense a light as in my suggestion, whereby the iron is introduced as a thin wire running through the core of the carbons.

The results in our experience have been already dealt with in a former article by Dr. A. J. Harrison and myself, so I will not now take up more space by referring to them. My experience with the light in lupus erythematosus is not encouraging, though in one case I have hopes that the X-rays have completely eradicated this disease. This, however, I freely admit was performed by inducing a severe X-ray "burn," which on healing left a pale white scar, without any sign of the disease on the site of the burn. In the remaining foci I have succeeded also in inducing a slight burn, and trust that when this is healed the disease will have been completely cured. But in this time can alone prove the value of the treatment.

NOTES RELATIVE TO FIGURES.

FIG. 1. Author's lamp with iron-cased carbon electrodes and water cooler as used as a hand lamp.

FIG. 2. Showing the arrangement of the electrodes.

FIG. 3. The same lamp used at the end of an adjustable bracket. Its own weight supplies the necessary pressure for rendering the part treated exsanguine. The source of electricity is the city mains, the current being used through a step-up transformer and condenser.

FIG. 4. Photograph reproduced by passing the light of an arc lamp through the chest of a thin boy on which a negative had been carefully strapped, the sensitive film having been fixed to the negative. All extraneous light was carefully excluded : only light passing through the body could reach the negative and so affect the film. Time, 3 minutes. Distance from arc, 12 ins. (circ).

## Figures and Tables

**Fig. I. f1:**
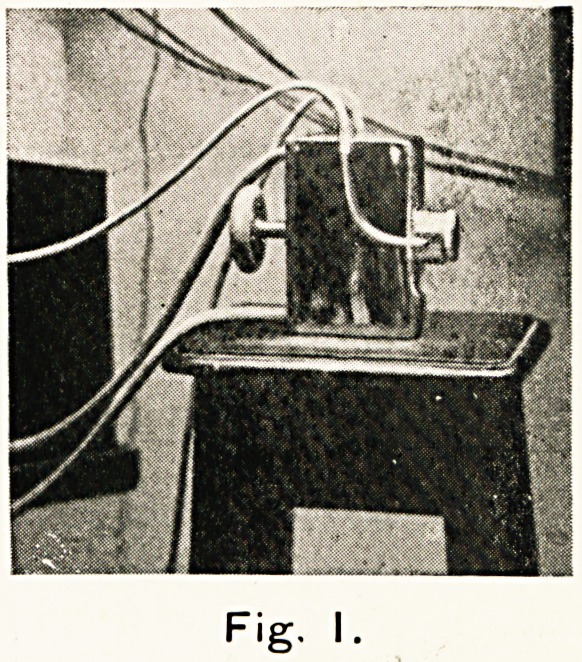


**Fig. II. f2:**
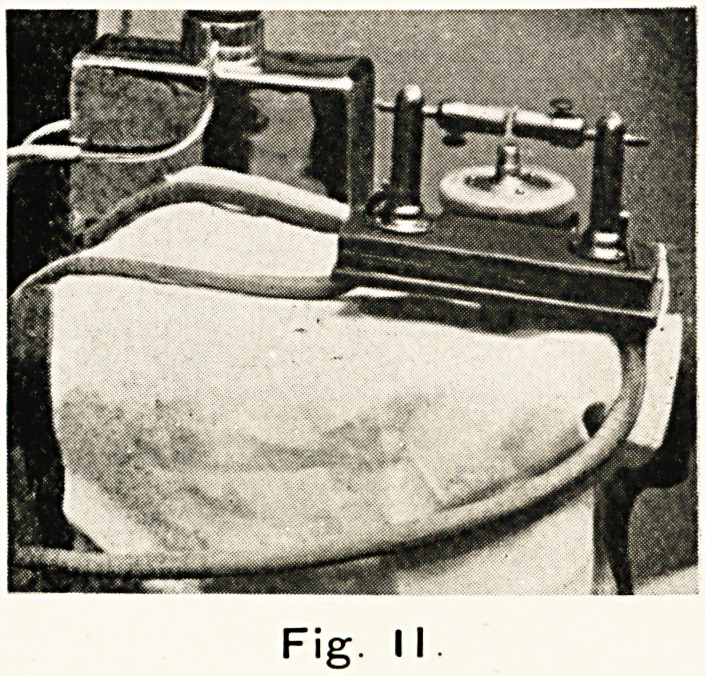


**Fig. III. f3:**
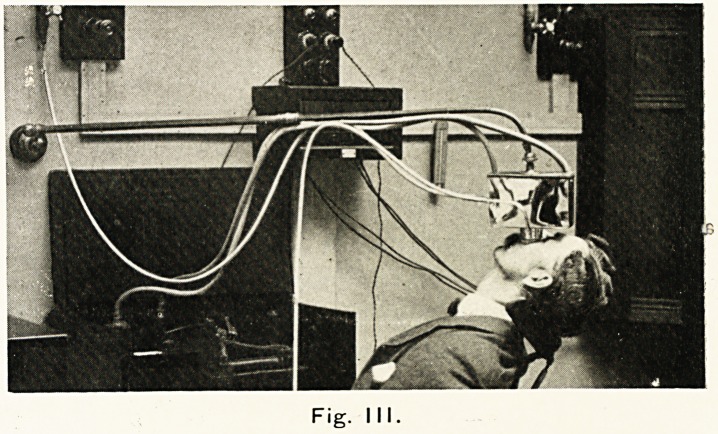


**Fig. IV. f4:**